# *PAX3* mutations and clinical characteristics in Chinese patients with Waardenburg syndrome type 1

**Published:** 2010-06-22

**Authors:** Juan Wang, Shiqiang Li, Xueshan Xiao, Panfeng Wang, Xiangming Guo, Qingjiong Zhang

**Affiliations:** State Key Laboratory of Ophthalmology, Zhongshan Ophthalmic Center, Sun Yat-sen University, Guangzhou, P.R. China

## Abstract

**Purpose:**

To detect paired box gene 3 (*PAX3*) mutations and associated phenotypes in Chinese patients with Waardenburg syndrome type 1 (WS1).

**Methods:**

Five unrelated families with suspected WS1 were selected from our Genomic DNA Repository for Hereditary Eye Diseases. The coding and adjacent intronic regions of *PAX3* were amplified by polymerase chain reaction and the amplicons were then analyzed by cycle sequencing. Variations detected were further evaluated in available family members as well as one hundred controls with heteroduplex-single strand conformational polymorphism (heteroduplex-SSCP) analysis and/or clone sequencing.

**Results:**

Three novel and two known mutations in *PAX3* were detected in five patients, respectively: c.567_586+17del (p.Asp189_Gln505delinsGluGlyGlyAlaLeuAlaGly), c.456_459dupTTCC (p.Ile154PhefsX162), c.795_800delCTGGTT (p.Trp266_Phe267del), c.799T>A (p.Phe267Ile), and c.667C>T (p.Arg223X). Two novel mutations proved to be de novo as their parents did not carry the mutations. All five patients with *PAX3* mutations had dystopia canthorum and different iris color and fundi between their two eyes. However, none had white forelock, skin hypopigmentation, and deafness.

**Conclusions:**

Our findings expand the frequency and spectrum of *PAX3* mutations and ethnic-related phenotypes in Chinese patients with WS1. De novo mutations in *PAX3* have not been reported before.

## Introduction

Waardenburg syndrome (WS) is an inherited disorder characterized by varying degrees of hearing loss and pigmentary anomalies affecting the eye, hair, and skin [1-6]. WS is clinically heterogeneous and has been classified into four major types and 10 subtypes as listed in Table 1 [5,7-17]. WS type 1 (WS1, OMIM 193500) and type 2 (WS2) are more common than type 3 (WS3) and type 4 (WS4). Overall, the syndrome affects perhaps 1 in 42,000 people [6].

**Table 1:** Classification of Waardenburg syndrome.

			**Phenotypes**						
**Types**	**OMIM**	**Inheritance**	**1**	**2**	**3**	**4**	**5**	**Genes or loci**	**Reference**
WS1	193500	AD	+	+/−*	+	-	-	PAX3	[8]
WS2	** **	** **	+	+/−**	-	-	-	** **	** **
WS2A	193510	AD	+	+	-	-	-	MITF	[9]
WS2B	600193	AD	+	+	-	-	-	1p21-p13.3	[17]
WS2C	606662	AD	+	+	-	-	-	8p23	[10]
WS2D	608890	AR	+	+	-	-	-	SNAI2	[11]
WS2E	611584	AD	+	+	-	-	-	SOX10	[12]
WS3	148820	AD or AR	+	+	+	+	-	PAX3	[13]
WS4	** **	** **	+	+	-	-	+	** **	** **
WS4A	277580	AR or AD	+	+/−	-	-	+	EDNRB	[14]
WS4B	613265	AR or AD	+	+	-	-	+	EDN3	[15]
WS4C	613266	AD	+	+	-	-	+	SOX10	[16]

Note: Phenotype 1: pigmentary disturbance of skin, hair and iris; Phenotype 2: Deafness; Phenotype 3: dystopia canthorum; Phenotype 4: upper limb abnormalities; Phenotype 5: aganglionic megacolon. The asterisk indicates presence in about 1/5 cases and the double asterisk indicates presence in about 3/4 cases.

Except for auditory-pigmentary disorder, dystopia canthorum is the typical phenotype of WS1 (Table 1). Mutations in the paired box gene 3 (*PAX3*, OMIM 606597) have been identified to be responsible for WS1 [18,19]. *PAX3* encodes a member of the mammalian PAX family of transcription factors, which contains two highly conserved domains for DNA binding, paired box domain and paired-type homeodomain [20]. Alternative splicing of *PAX3* results in several different-length transcripts, of which the longest transcript contains 10 exons, and consequent proteins with distinct carboxyl termini [21]. PAX3 plays a regulatory role in the early embryonic development of the pigment system [22] and is required to expand a pool of committed melanoblasts or restricted progenitor cells early in development [23]. Heterozygous mutations in *PAX3* have been reported in familial and sporadic WS1, while heterozygous or homozygous mutations have been detected in patients with WS3 [8,13,24,25]. Although many mutations have been identified in Caucasians, several cases have been determined in the Chinese population [26,27]. Fundus changes for WS1 patients with *PAX3* mutations have not been reported.

In the present study, five mutations in *PAX3*, including three novel ones and two known ones, were identified in five unrelated Chinese families with WS1. All patients with the 5 mutations presented dystopia canthorum and different colors of the irises and fundi but none of those showed visible pigmentary changes on their hair and skin, indicating an ethnic specific phenotypes.

## Methods

### Patients

Five unrelated patients were recruited from our Pediatric and Genetic Eye Clinic, Zhongshan Ophthalmic Center, Guangzhou, P.R. China. Diagnosis of WS1 was based on criteria previously described [4,28]. Informed consent conforming to the tenets of the Declaration of Helsinki and following the Guidance of Sample Collection of Human Genetic Diseases (National 863-Plan) by the Ministry of Public Health of China was obtained from participating individuals before the study. All participants received detailed ophthalmological examinations performed by ophthalmologists (Q.Z. or X.G.). Unrelated controls (100) were collected from normal volunteers. This study was approved by the Institutional Review Board of Zhongshan Ophthalmic Center.

### Variation analysis

Genomic DNA was isolated from venous leukocytes. Genomic fragments encompassing coding regions and adjacent intronic regions of *PAX3* were amplified by polymerase chain reaction (PCR), using eleven primer pairs (*PAX3*: NCBI human genome build 36.1, NC_000002.11, NM_181459.2, NP_852124.1), including previously reported primers for exons 1–9 [26] and two new primer pairs for exon 10 (Table 2). The amplicons from individual exon were purified and analyzed by cycle sequencing with ABI BigDye Terminator Cycle Sequencing Kit v3.1 (ABI Applied Biosystems, Foster City, CA) on an automatic DNA sequencer (ABI 3100 Genetic Analyzer, Applied Biosystems). Sequencing results from patients as well as the consensus sequences from the NCBI Human Genome Database were imported into the SeqManII program of the Lasergene package (DNAStar Inc., Madison, WI) and aligned to identify variations. Each variation was confirmed by bidirectional sequencing. Variations were named following the nomenclature recommended by the Human Genomic Variation Society (HGVS).

**Table 2:** Primers for amplifying and sequencing *PAX3* genomic fragments.

**Exon**	**Forward Primers(5′-3′)**	**Reverse Primers(5′-3′)**	**Product size (bp)**	**Annealing temperature (°C)**
PAX3-ex1	TCACCACAGGAGGAGACTCA	GAGGCCCTCCCTTACCTTC	472	60
PAX3-ex2	TACGTGCTGCTGTTCTTTGC	TTACGCACCTTCACAAACCTC	442	60
PAX3-ex3	TCTGGTCTGCCCCTTTCTAA	ATTGGGGTGATTACGTCTGG	388	60
PAX3-ex4	GCTGGAGAAGGATGAGGATG	CTCCAAGTGACCCAGCAAGT	351	60
PAX3-ex5	TGTCTTGCAGTCGGAGAGAG	GGTGGACTTCTGTGTGTCGT	492	60
PAX3-ex6	AATTCGCCCAAACAACACA	CAGAGAAATCGCCTGGAAGT	368	60
PAX3-ex7	TGGCGATGAACTTTTGCAC	GGGTGGAGAGAAAGGAAACC	451	60
PAX3-ex8	TCGTCGGGCATGATGTAATA	AGGAGAAATTGCCCCCTAAA	359	60
PAX3-ex9	GAATTGTCCCAGCATGACCT	TGCTCCAGGTCTTCCTCTTC	311	62
PAX3-ex10a	ACTGGCCCTGTTTCTGGTCT	TGGCAAACATCACTGCACTC	943	60
PAX3-ex10b	CCAGTTCACATTTATTTGG	CTCATAGAAAGGGTCCAC	887	60

Any variation detected by sequence analysis was further evaluated in 100 controls by heteroduplex-SSCP analysis. In addition, one multiple-nucleotide deletion was further analyzed by clone sequencing, using the method we described previously [29]. NNSPLICE version 0.9 was used to predict splice sites.

## Results

### Clinical phenotype

**Figure 1:**
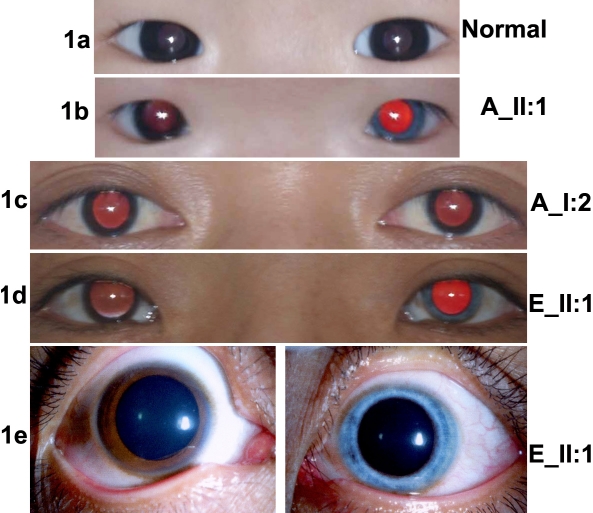
Photographs of eyes from WS1 patients and controls. **A**: Baby with normal iris pigmentation and normal facial characteristics. **B**: 15-month-old girl (II:1 from family A in Figure 3) with dystopia canthorum, heterochromia iridis, broad nasal root, and a horizontal distance between the inner canthi of 28 mm. **C**: Normal adult (I:2 from family A). **D**, **E**: Adult II:1 from family E showed dystopia canthorum, heterochromia iridis (left iris).

**Figure 2:**
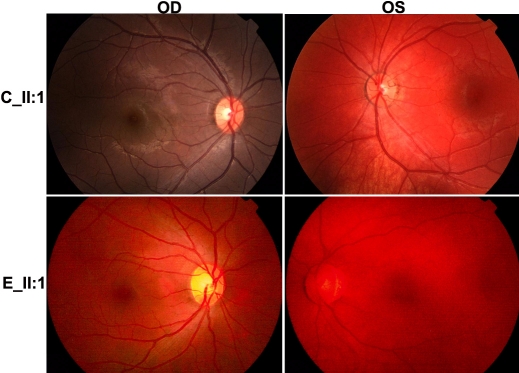
Photographs of fundi from WS1 patients with *PAX3* mutations. Fundus photos were taken from the right (OD) and the left (OS) eyes of two patients, II:1 from family C and II:1 from family E. The colors of fundus photos were different between two eyes in both patients, where mild retinal hypopigmentation was demonstrated in the left eyes of both patients. The difference of fundus colors between C_II:1 and E_II:1 is of no clinical significance as different fundus cameras were used. Except for hypopigmentation, the fundus structure was comparatively normal in the patients.

The most significant sign in all five unrelated patients is different colors between two eyes, which resulted from heterochromia iridis (Figure 1, Table 3). All patients had dystopia canthorum (Figure 1). Ocular fundus examination revealed different colors between two fundi (Figure 2), which have not been described before. In all 5 patients the eye with generalized iris hypopigmentation also had mild retinal hypopigmentation. In the eyes with pigmentary changes, however, the fundus vessel distribution, macular architectural and visual acuity seemed to be normal (Figure 2 and Table 3). None of the 5 patients had pigmentary changes on their skin, hair, eyebrows, and eyelashes, which are the common signs in Caucasian patients. Deafness was not observed in three patients while the hearing function could not be measured in the other two babies. Anomalies on limb development were not observed in all 5 patients.

**Table 3:** Clinical findings in patients from Families A-E and mutations identified in *PAX3*.

** **	** **	** **	**Visual acuity**		** **	** **	** **	** **	** **	** **	** **
**ID**	**Sex**	**Age (yrs)**	**OD**	**OS**	**Mutation**	**Effect**	**Differently colored eyes**	**Fundus hypopigmentation**	**Dystopia canthorum**	**Deafness**	**Family history**
A-II:1	F	1	NA	NA	c.567_586+17del	p.Asp189_Gln505delinsGluGlyGlyAlaLeuAlaGly	OS	OS	Yes	NA	No
B-II:1	M	0.6	NA	NA	c.456_459dupTTCC	p.Ile154PhefsX162	OS	OS	Yes	NA	No
C-II:1	M	7	1.00	0.90	c.795_800delCTGGTT	p.Trp266_Phe267del	OS	OS	Yes	No	No
D-IV:1	M	6	0.90	1.00	c.799T>A	p.Phe267Ile	OD	OD	Yes	No	Yes
E-II:1	F	23	1.50	1.50	c.667C>T	p.Arg223X	OS	OS	Yes	No	No

Note: NA: Not available because they are too young. None of the 5 probands had white forelock and skin hypopigmentation.

### Variation detection

**Figure 3:**
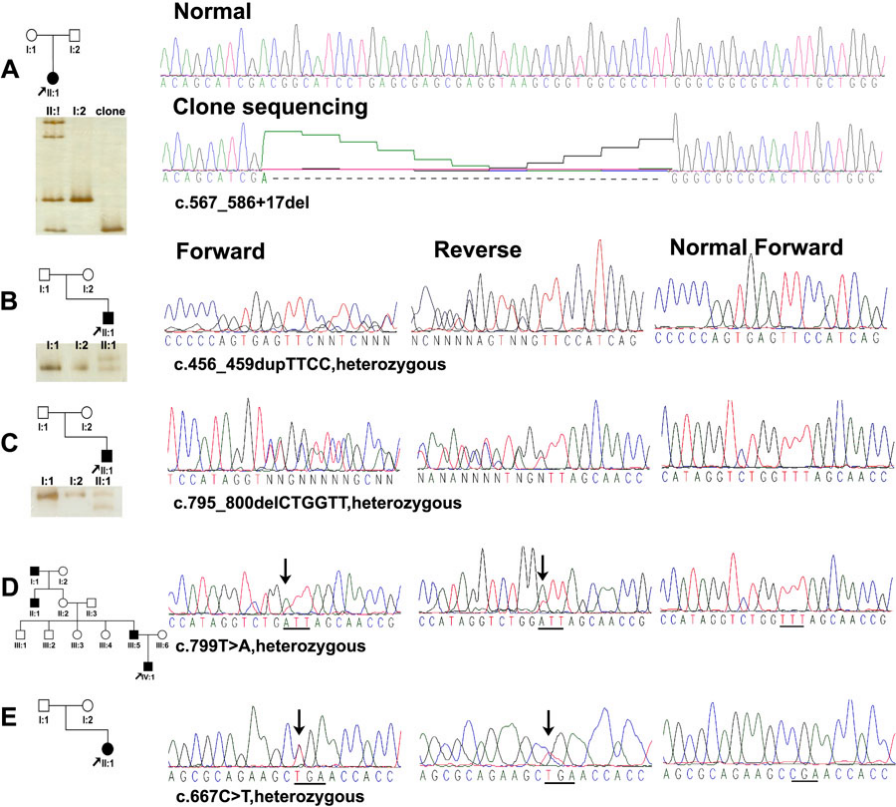
Pedigrees and sequence chromatography. Black filled symbols represented individuals affected with WS1 in each family. Arrow indicated the proband in each family. **A**: Clone sequencing demonstrated a c.567_586+17del mutation in Family **A**. For the other four families, bidirectional sequencing results were shown for the regions with variations. Underline below the sequence highlighted the codon affected by the mutation. Gel electrophoresis band patterns below the pedigrees of families **A**, **B**, and **C** were the results of heteroduplex-SSCP analysis, which demonstrated the presence and absence of three novel mutations in other family members. The c.456_459dupTTCC and c.795_800delCTGGTT mutations in the probands from families **B** and **C** were not detected in their parents, suggesting de novo mutations. The c.567_586+17del mutation in Family **A** was not present in the patient’s father but sample from her mother was not available. For families **D** and **E**, genomic samples from other family members were not available.

In the 5 patients, five heterozygous mutations in *PAX3* were detected, including c.567_586+17del (p.Asp189_Gln505delinsGluGlyGlyAlaLeuAlaGly), c.456_459dupTTCC (p.Ile154PhefsX162), c.795_800delCTGGTT (p.Trp266_Phe267del), c.799T>A (p.Phe267Ile), and c.667C>T (p.Arg223X; Table 3, Figure 3). The first three mutations were novel and, therefore, were further confirmed by heteroduplex-SSCP analysis (Figure 3). The other two mutations were known mutations. All five mutations were absent in 100 normal controls based on heteroduplex-SSCP analysis (data not shown).

The c.567_586+17del mutation was identified in a baby from Family A (A-II:1). Direct sequencing revealed a heterozygous variation involving multiple nucleotides in exon 4 region. Cloning sequencing revealed a 37 bp deletion affecting both exon 4 and intron 4 (Figure 3A). A new splice site is predicted to be created downstream by NNSPLICE. The encoded protein would be truncated.

The c.456_459dupTTCC and c.795_800delCTGGTT mutations were only present in the probands (Figure 3, B-II:1, C-II:1) but not in their parents, demonstrating de novo mutations that have been rarely reported in *PAX3*.

## Discussion

In this study, three novel and two known mutations in *PAX3* were identified in five unrelated Chinese patients. The three novel mutations would result in frameshift or inframe deletion if transcribed and translated, suggesting putative disease-causing. Unilateral sapphire iris with pink pupil and retinal depigmentation as well as dystopia canthorum without other abnormalities suggested a diagnosis of WS1.

Of the five *PAX3* mutations, three were novel (c.567_586+17del, c.456_459dupTTCC and c.795_800delCTGGTT) and the other two were previously reported (c.799T>A and c.667C>T) [30,31]. Two novel mutations, c.456_459dupTTCC and c.795_800delCTGGTT, were proved to be de novo as their parents did not carry the mutations, suggesting that natural occurring new mutations in *PAX3* of the Chinese population is not uncommon. Based on available information, de novo mutations in *PAX3* have rarely been mentioned before. Three of the five mutations, c.567_586+17del (p.Asp189_Gln505delinsGluGlyGlyAlaLeuAlaGly), c.456_459dupTTCC (p.Ile154PhefsX162) and c.667C>T (p.Arg223X), are predicted to encode premature truncated proteins affecting the paired-type homeodomain [20]. The other two mutations, c.795_800delCTGGTT (p.Trp266_Phe267del) and c.799T>A (p.Phe267Ile), would also affect the paired-type homeodomain, if translated.

Clinical manifestation of the 5 Chinese patients with *PAX3* mutations is consistent with the phenotypes of WS1. However, pigmentary changes on skin, hair, eyebrows, and eyelashes are absent in these Chinese patients, indicating an ethnic specific variations in clinical expression. Fundus hypopigmentation in WS1 patients have been demonstrated in the Chinese patients. Although fundus hypopigmentation was recorded in WS in previous reports, it has not been described in WS1 patients with *PAX3* mutations before. Understanding the typical and atypical phenotypes of Chinese WS1 patients is of clinical importance as such patients may be misdiagnosed as unilateral ocular albinism, especially since mild dystopia canthorum is not uncommon in Southern Chinese population.
